# Evaluation of the Erector spinae plane block for postoperative analgesia in laparoscopic ventral hernia repair: a randomized placebo controlled trial

**DOI:** 10.1186/s12871-024-02566-x

**Published:** 2024-05-29

**Authors:** Marie Sørenstua, Johan Ræder, Jan Sverre Vamnes, Ann-Chatrin Linqvist Leonardsen

**Affiliations:** 1 Department of Anesthesia, Kalnesveien 300, Grålum, 1714 Norway; 2 Institute of Clinical Medicine, University of Oslo, Oslo, Norway; 3 Faculty of Health, Welfare and Organisation, Ostfold University College, Fredrikstad, Norway; 4 Department of Anesthesia, Ostfold Hospital Trust, Kalnes, Norway

**Keywords:** Acute pain, Anesthesia, Postoperative analgesia, Regional anesthesia

## Abstract

**Background:**

The Erector spinae plane block (ESPB) reduces postoperative pain after several types of abdominal laparoscopic surgeries. There is sparse data on the effect of ESPB in laparoscopic ventral hernia repair. The purpose of this study was to test the postoperative analgesic efficacy of an ESPB for this procedure.

**Methods:**

In this prospective, double-blind, randomized controlled study, adult patients undergoing laparoscopic ventral hernia repair were randomly assigned to either bilateral preoperative ESPB with catheters at the level of Th7 (2 × 30 ml of either 2.5 mg/ml ropivacaine or saline), with postoperative catheter top ups every 6 h for 24 h. The primary outcome was rescue opioid consumption during the first hour postoperatively. Secondary outcomes were total opioid consumption at 4 h and 24 h, pain scores, nausea, sedation, as well as Quality of Recovery 15 (QoR-15) and the EuroQol-5 Dimensions (EQ-5D-5L) during the first week.

**Results:**

In total, 64 patients were included in the primary outcome measure. There was no significant difference in rescue opioid consumption (oral morphine equivalents (OME)) at one hour postoperatively, with the ESPB group 26.9 ± 17.1 mg versus 32.4 ± 24.3 mg (mean ± SD) in the placebo group (*p*= 0.27). There were no significant differences concerning the secondary outcomes during the seven-day observation period. Seven patients received a rescue block postoperatively, providing analgesia in five patients.

**Conclusion:**

We found no difference in measured outcomes between ESPB and placebo in laparoscopic ventral hernia repair. Future studies may evaluate whether a block performed using higher concentration and/or at a different thoracic level provides more analgesic efficacy.

**Trial registration:**

NCT04438369; 18/06/2020.

**Supplementary Information:**

The online version contains supplementary material available at 10.1186/s12871-024-02566-x.

## Introduction

The Erector spinae plane block (ESPB) was first introduced by Forero in 2016 [1] as a novel truncal block. Since then, the ESPB has been utilized for analgesia in breast surgery, thoracic and cardiac surgery, abdominal surgery, spine surgery, as well as upper and lower limb surgery [2–5]. The ESPB has a low-risk safety profile and is deemed as a simple block to perform [6]. Recent studies into the mechanism behind the ESPB, show consistent spread of LA to the paravertebral space and the ventral rami of the spinal nerves in live subjects [7, 8]. Thus, the block may have the potential to provide visceral and somatic analgesia comparable to a paravertebral block [7–9].

Ventral hernia repair patients will often have to stay in the hospital because of severe postoperative pain and significant opioid consumption during the first 24 h even though the surgery itself is laparoscopic [10, 11]. The mesh used in the procedure is fastened with helical tacks to the peritoneum and abdominal wall and is the cause of the significant postoperative pain experienced by these patients [12].

To our knowledge, there is only one case series with four patients suggesting some effect of ESPB on postoperative pain relief in ventral hernia repair [11].

This study aimed to assess the analgesic efficacy of bilateral Th7 ESPB blocks in patients undergoing elective laparoscopic ventral hernia repair.

## Methods

The study had a prospective, double-blinded randomized controlled study design, with two study arms. The study was approved by the Norwegian Regional Committee for Medical and Health Research Ethics (ref. no. 2018/2239). The trial was registered at Clinicaltrials.gov on the 18/06/2020 (NCT04438369) before patient enrolment. The manuscript adheres to the Consolidated Standards of Reporting Trials guidelines (CONSORT 2010).

### Setting and participants

The study was performed at a county hospital with a catchment area of approximately 320.000 inhabitants. Patients scheduled for an elective laparoscopic ventral hernia repair and fulfilling the inclusion criteria were invited to participate. Eligible patients received written and oral information from an anesthesiologist prior to inclusion, and a signed informed consent form was obtained. The inclusion criteria were: age > 18 years, Body Mass Index (BMI) 18.5–40, weight > 65 kg, American Society of Anesthesiologists (ASA) classification I-III, and a planned hospital stay > 24 h. Exclusion criteria were: inability to speak or understand the Norwegian language; inability to adhere to the protocol; allergy to latex, local anesthesia, non-steroidal anti-inflammatory drugs or opioids; chronic pain prior to surgery demanding daily opioids; addiction to medication or alcohol; liver or kidney failure; local infection at the site of injection; systemic infection; atrioventricular (AV) block 2–3; Diabetes (insulin-treated) and pregnancy.

### Intervention

All patients received pre-operative multimodal analgesia consisting of oral paracetamol 2 g and diclofenac 100 mg at least one hour before the start of surgery. Reduced doses of 1.5 g and 50 mg respectively were given if weight < 70 kg or age > 70 years as per hospital standard.

**Fig. 1:**
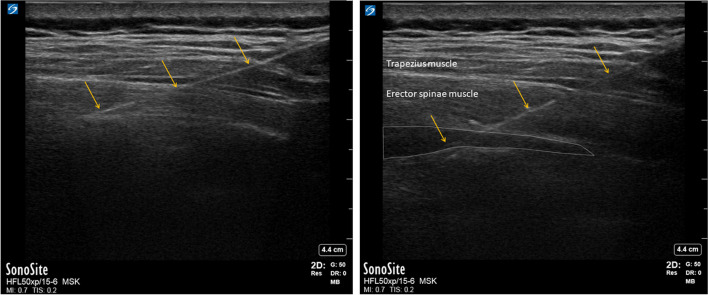
Ultrasound image of an ESPB block. Yellow arrows indicate needle trajectory and needle contact with the transverse process. The white dotted area represents LA injected underneath the Erector spinae muscles

All patients were monitored with standard ASA surveillance (SpO2, NIBP, ECG, and ETCO2). All patients were taken to the post-anesthesia care unit (PACU) prior to surgery. They were offered sedation with midazolam 1- 2 mg and/or alfentanil 0.5 -1.0 mg before receiving the block. The skin on the posterior truncus was prepared thrice with chlorhexidine 5 mg/ml with added phenol red. All interventions were performed in a sitting position under ultrasound guidance (Fujifilm, Sonosite, X-porte ultrasound system, Bothell, Washington, USA). The level of the Th7 transverse process was determined and marked before the start of the intervention by counting the ribs cranially from the 12th rib using the curvilinear probe. A linear probe (Sonosite HFL50xp) or curvilinear probe (Sonosite C60XP), covered with a sterile cover, was used depending on the depth of the transverse process (under or over 4 cm). After identifying the transverse process, the catheter needle (E-Cath PLUS Tsui, 18 G, 83 mm, PAJUNK GmbH, Geisingen, Germany) was inserted from cranial to caudal using an in-plane technique and a parasagittal view. After contacting the transverse process with the needle, the correct visual spread underneath the Erector spinae muscles was confirmed by injecting up to 10 ml saline (Fig. 1).

A nurse injected 30 ml of study medication of either ropivacaine 2.5 mg/ml ropivacaine or saline, through the catheter while the anesthesiologist continuously confirmed the correct spread visually. The internal catheter that extends 1 cm beyond the external catheter tip was inserted and fixated. The process was repeated on the contralateral side. The patients were tested for loss of sensation to cold and pinprick by another anesthesiologist blinded to the intervention 30 min after the intervention. Testing of cutaneous loss of sensation to cold was performed with a latex glove containing ice cubes, while loss of cutaneous sensation to pinprick was tested using an 18G short bevel needle. Any decrease in heart rate and blood pressure combined with clinical symptoms was treated with 0.5 mg atropine.

General anesthesia was provided with target-controlled infusions (TCI) of propofol and remifentanil titrated to clinical response. Endotracheal intubation was performed after endotracheal spray with 50 mg of lidocaine, according to local procedures. No muscle relaxant was used. All patients received ondansetron 4 mg, dexamethasone 8 mg, and oxycodone 5 mg iv before the completion of surgery.

Prior to the insertion of the three abdominal ports, the surgeon infiltrated a total of 20 ml 2 mg/ml ropivacaine subcutaneously at the port sites. A maximum intra-abdominal pressure of 12 mmHg was used for CO_2_ inflation. The hernia was repaired using mesh nets adjusted to the size of the hernia fixated with tacks to the peritoneum.

In the PACU, all patients received a PCA pump programmed with an intravenous bolus dose (0.03 mg/kg) of oxycodone. The lockout time was 5 min and the maximum allowed number of boluses per hour was 8. There was no continuous infusion. The patients were instructed to take a bolus dose if their NRS was ≥ 4 at rest. At one hour postoperatively all included patients were scored according to the outcome measures. If any patient had a pain score of NRS > 7, a sedation score > 3, and an opioid consumption > 45 mg OME at this time, they were given a rescue ropivacaine bolus of 2 × 30 ml of 2 mg/ml in their ESPB-catheters, and subsequently 2 × 30 ml every six hours for the rest of their stay. For a patient in the intervention group, this meant an expedited first bolus and an adjustment of the timing of the following doses. From this point on, the investigators, nurses and the patients themselves knew that ropivacaine was given, but the original group allocation was not revealed, and the patients were scored as originally planned.

The patients were transferred back to the ward after a minimum of one hour, where they stayed for a minimum of twenty-four hours before being discharged. In the ward, all patients received multimodal analgesia consisting of oral paracetamol 1000 mg four times daily and diclofenac 50 mg three times daily. The ward nurses administered a bolus dose of 30 ml of the study medication in both erector spinae catheters (total of 60 ml) every 6 h to a total of 4 times in all patients. The intervention group received 2 mg/ml ropivacaine and the placebo group 0.9% saline solution. The PCA pump and catheters were discontinued after twenty-four hours after their arrival in the PACU.

After discharge, the patients were instructed to take paracetamol 1000 mg four times daily for the first three days, adding codeine 30 mg if needed. Further pain medication was at the patient’s discretion and the patients were instructed to register the amount and type of medication. A study nurse contacted the patients by telephone after 24 h, 48 h, and 7 days, and asked for the outcome measures.

### Primary outcome

The primary outcome measure was the total opioid consumption during the first hour postoperatively, as measured by oral morphine equivalents (OME), using an opioid conversion table (Supplemental material) [13]. In this conversion table, 1 mg oxycodone iv corresponds to 3 mg OME.

### Secondary outcomes

Secondary outcomes were total opioid consumption at 4 and 24 h, as well as pain scores (NRS 0–10) at rest and when coughing, nausea measured with the Postoperative nausea and vomiting (PONV) impact scale [14], and the level of sedation measured by the Pasero Opioid-Induced Sedation Scale (POSS) [15], measured by a nurse investigator at the time points 1, 2, 3, and 4 h. Further secondary outcomes were the Quality of recovery (QoR-15) score [16] as well as the complementary EuroQol (EQ-5D-5L) score [17] and NRS at rest measured at 24 h, 48 h and 7 days postoperatively. The QoR-15 measures different aspects of the patient's perceived postoperative recovery as five dimensions of health; patient physiological support, comfort, emotional state, physical independence, and pain [18]. The EQ-5D-5L describes the five health dimensions; mobility; self-care; usual activities; pain/discomfort; and anxiety/depression. These five health dimensions are then divided into 5 levels and were evaluated in this study using the EQ-5D-5L Devlin value set for England [19]. The study nurse registered the 24 h, 48 h and 7 day measurements by telephone contact with the patients.

### Sample size calculation

The sample size was calculated from our series of 20 pilot patients on ventral hernia surgery without block, who needed on average 24.6 mg OME rescue analgesia during the first hour, SD = 17.35 mg. In a two-sided test, and to show a 50% reduction in rescue OME consumption after a successful block with 80% power and 0.05 as level of significance, at least 2 × 33 patients should be included, a total of 66 patients. To adjust for missing data and protocol violations we decided to include 2 × 35 patients to a total of 70 patients.

### Randomization and blinding

The randomization was done through a computer-generated block randomization process (randomization.com) to reduce bias [20]. The block sizes were randomly chosen to be 2, 4, and 6. A study nurse prepared and placed the notes of study allocation covered with aluminum foil into opaque envelopes. The envelopes were numbered from 1- 70. Two PACU nurses opened the envelope and drew up the allocated study medication in unmarked syringes. These nurses did not take any part in the treatment or evaluation of the patients, to make all personnel responsible for medication and registrations blinded. The anesthesiologist performing the block was blinded to the allocation. Unmarked containers of the allocated study medication, except from the study patient number, were delivered to the ward.

### Statistical analysis

Statistical analyses were conducted using STATA Version 16 [21]. Continuous data with normal distributions after Shapiro–Wilk normality tests are presented as means (SD or range as appropriate), and non-normally distributed data and ordinal data are presented as medians with interquartile ranges [25–75]. Comparisons of baseline statistics were performed with unpaired t-tests and Mann–Whitney tests as appropriate. A multiple regression analysis with intervention group, age, and BMI as cofactors was performed to test associations with the primary outcome. A multilevel mixed-effects linear regression model was used to analyze repeated measurements (pain, OME consumption, global QoR-15, and EQ-5D scores). The model included time, treatment, and the interaction between the two as fixed effects, as well as the patient indicator and residual variance as random effects. Any significant differences between the groups at any time point were calculated using a linear combination of parameters (lincom). To analyze differences in nausea and sedation, the repeated-measures ANOVA was utilized because the mixed model did not converge.

## Results

**Fig. 2:**
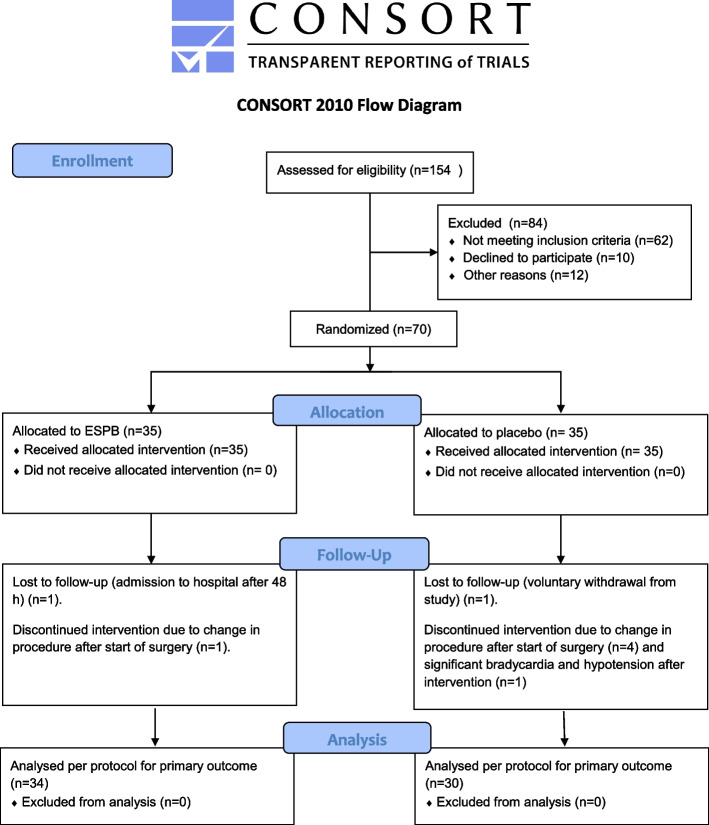
CONSORT flow diagram

Patients were enrolled from 01/09/2020 to 05/12/2023. Of 154 patients screened for eligibility, 70 patients were included and randomized, of which 64 completed the study protocol until the primary outcome. 6 patients were excluded after inclusion, 1 due to significant bradycardia and hypotension during the intervention, and 5 patients due to a change in procedure after the start of surgery. Two patients were lost to follow-up at 48 h and 7 days. One due to admission to the hospital two days postoperatively, and one because of withdrawal from the study. See the CONSORT flow diagram (Fig. 2).

There were no significant differences between the groups concerning demographic data: age, weight, body mass index (BMI), gender, ASA classification, surgical duration, or intraoperative medication. Table 1 presents an overview of patients’ characteristics and per-operative information.

**Table 1:** Patient Characteristics

Patient Characteristics	ESPB (*N* = 33)	Placebo (*N* = 30)	*P*-value
Age (SD)	60.9 (12.9)	62.1 (12.95)	0.66
Weight (range)	90.8 (67–120)	87.4 (65–125)	0.31
BMI (range)	30.5 (25.1–37)	30.4 (23.0–38.6)	0.89
ASA (I/II/III)*	3/24/6	7/18/5	0.29
Operation time [IQ]	40 [31–61]	37 [30–60]	0.70
Propofol [IQ]	658 [513–820]	616 [491–742]	0.59
Remifentanil [IQ]	745 [575–1205]	657.5 [545–1005]	0.38

Age: in years. *SD *standard deviation. Weight; in kilograms. *BMI* body mass index kg/height(m^2^). ASA* The American Society of Anesthesiologists (ASA) criteria; normal health (I), mild systemic disease (II), severe systemic disease (III), severe systemic disease that is a constant threat to life (IV), moribund patient (V). Operation time; in minutes. *IQ* interquartile range 25–75. Propofol/Remifentanil; in milligrams/in micrograms. The data are represented as means (SD/range as appropriate) for continuous data with normal distribution, and median [IQ 25–75] for non-normally distributed data. Comparisons of baseline statistics were performed with unpaired t-tests for the normally distributed data and Mann–Whitney tests for the non-normally distributed data

### Outcomes

#### Primary outcome

The mean OME consumption at 1 h postoperatively was similar in the two groups, in the ESPB group 26.9 ± 17.1 (mean ± SD) versus 32.4 ± 24.3 mg in the placebo group (*p*= 0.27, 95% CI [-16.3, 4.66]).

#### Secondary outcomes

**Fig. 3:**
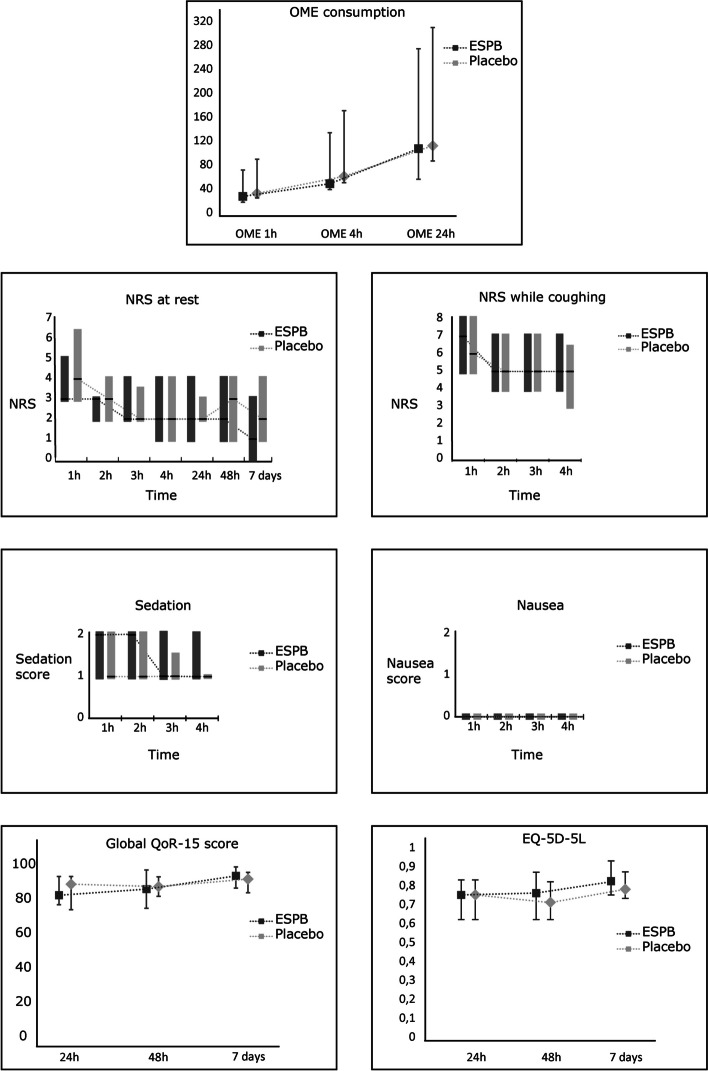
OME = Oral morphine equivalents presented in milligrams (mg). h = hours. Numerical rating scale (NRS). The data are represented as means with SD for continuous data with normal distribution (OME consumption), and median (interquartile range 25–75) for ordinal data (NRS, sedation, nausea, QoR-15 and EQ-5D). P-values are calculated with linear combination of parameters (lincom) using the results derived from a repeated measures mixed model. The group p-value for nausea and sedation are calculated using a repeated measures ANOVA

There were no significant differences in OME consumption, pain, nausea, sedation, QoR-15 or EQ-5D-5L scores in any registration during the seven-day observation period (Fig. 3).

**Fig. 4:**
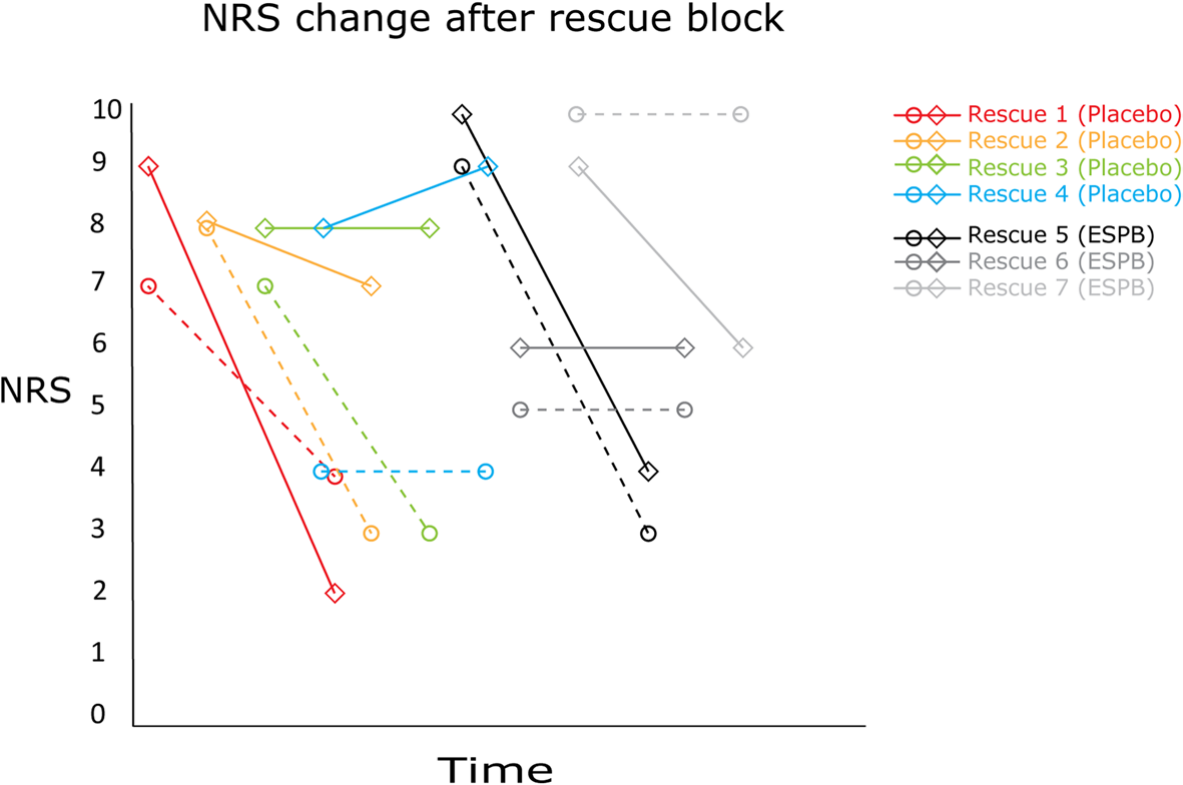
The change in NRS at rest (circle with dotted line) and NRS when coughing (diamond with solid line) from 1 to 4 h after a rescue block at 1 h. The patients are marked as ESPB group or placebo group

A rescue block was performed on 7 patients (4 in the control group and 3 in the intervention group) one hour postoperatively, on the basis of the ethical considerations of not leaving patients in severe pain without adequate pain relief from high opioid consumption. We observed an analgesic effect in 5 patients. Out of these, 2 patients had a reduction of > 3 in NRS in pain scores at rest and during coughing, as well as significantly reduced opioid consumption during the first 4 subsequent hours. In 2 patients there were a reduction in two of these parameters, while in 1 patient one parameter was reduced. In 2 patients there were no significant changes in pain scores nor opioid consumption. See Fig. 4 and Table 2.

**Table 2:** OME consumption after rescue block

	**0–1 h**	**1–4 h**	
Rescue 1	105 mg	0 mg	
Rescue 5	46.8 mg	7.8 mg	
Rescue 7	51.6 mg	10.2 mg	

Significant opioid consumption decrease (> 30 mg OME) was observed in 3/7 patients that received a rescue block at 1 h postoperatively. OME = oral morphine equivalents

Five patients received atropine due to bradycardia and hypotension during the intervention. One patient had to be excluded due to a bradycardic episode with loss of consciousness. No other adverse effects were recorded.

## Discussion

In this randomized, double-blinded study comparing the ESPB versus placebo in laparoscopic ventral hernia repair patients, we could not identify any significant differences in OME consumption or pain at 1 h, 4 h, and 24 h postoperatively, nor in any other observed outcome at any time during the seven-day observation period.

Our results are in line with a meta-analysis done on the postoperative effects of ESPB in patients after liver surgery that could not find any significant reduction of postoperative pain scores, nor any reduction of cumulative opioid consumption at 24 h [22].

However, our results contrast with results from a meta-analysis done on a composite of abdominal surgeries, where an ESPB was superior to a placebo block [2]. In other systematic reviews and meta-analyses for more specific abdominal interventions, the ESPB has also been shown to decrease postoperative pain scores, opioid consumption, and the incidence of PONV [23, 24].

Chin et al. presented a case series of four patients after laparoscopic ventral hernia surgery, where an ESPB placed at the level of Th7 showed promising effects on postoperative opioid consumption and pain [11]. Also, Abu Elyazed et al. did a randomized controlled trial performing a bilateral ESPB at Th7 in patients scheduled for open epigastric hernia repair (*n*= 60). Here, the authors showed significantly lower pain scores at 12 h, as well as lower median rescue opioid consumption [25].

We were not able to find any significant differences between the two main groups, but in the rescue patients, we observed a change in NRS > 3 and/or opioid consumption > 30 mg OME in 5 out of the 7 patients who received a rescue block after one hour. This may suggest that some patients experience pain relief from the block when the patients have a strong baseline pain, and even that a repeat block may help in the patients who already have received a block. The effect of an extra bolus in the group that already received a block may be due to an increase in LA volume and as a consequence a larger spread of the LA. These results should still be interpreted with caution as the number of patients is low, and the effects of rescue blocks were not a primary aim in the study design.

One reason for the lack of difference between the main groups may be a too cranial injection level. In a volunteer study performed by our group [8], we showed that an injection performed at the level of Th7 had a median cephalad spread to Th4 and a caudal spread to spread to Th8/9. Hence, one may deduce that the best effect of the block would be expected to be at the levels of Th4-Th8/9. As the umbilicus dermatomal level is around Th10 there is a possibility that nociception from the lower part of the net was not covered.

Other reasons may be that the concentration of the LA (2.5 mg/ml ropivacaine for the bolus dose and 2 mg/ml for the subsequent refill doses) was too low to provide adequate analgesia. In this study, the weight limit for inclusion was 65 kg to ensure that no patients received a total dose of LA that superseded the recommended max daily dose of 11 mg/kg/24 h [26]. In a clinical setting with patients who have an ideal weight above this weight, there is the possibility of increasing the concentration of the LA to possibly achieve better analgesia.

However, until proven otherwise in further studies, we must deduce from our data that the ESPB block is not efficient for analgesia in this type of surgery.

### Strengths and limitations

In this study all blocks were performed by a single experienced anesthesiologist, experienced surgeons participated, only one hospital was involved, and there were highly standardized criteria for all aspects of patient handling. All participants of the study, the patients, nurses, and the anesthesiologist were blinded to the intervention. All patients received a patient-controlled analgesia (PCA) pump, to standardize the need for opioid rescue medication.

A limitation of this study is the difference between patients as to the size of the nets placed and the number of net fixations. Logically a larger net would involve a larger part of the peritoneum and more significant pain. We were not able to control for the difference in net size, but this situation reflects clinical reality.

Another limitation of the study was the choice of the primary outcome after 1 h, not 24 h which is more common. The reason behind this choice was the expectance of a group of patients with severe postoperative pain needing intervention despite rescue opioid administration. In our hospital the standard treatment after laparoscopic ventral hernia surgery has been multimodal analgesia and opioid medication. The expectance of a group of patients that needed intervention arose from our previous clinical experience with these patients, where quite a few need a variant of a regional technique (Rectus sheet block, Quadratus lumborum block or an ESPB). By setting the primary outcome to 1 h we could still calculate a primary outcome containing these patients.

Also, our power analysis mandated that 66 patients should be included. Even though we included 70 patients, we lost 6 patients after inclusion. Still, the values of all outcome variables were very similar between the two groups, and would hardly be very different with a higher number of patients fulfilling all outcome measurements.

## Conclusions

We could not find any significant difference in opioid consumption, pain scores, nausea, sedation, Qor-15, or EQ-5D between patients who received a pre-and postoperative ESPB and patients who received a placebo block. This suggests that the ESPB is not effective for this specific procedure. However, further studies are needed to see whether a block performed at a lower thoracic level and/or with an increased concentration of LA would offer different results.

### Supplementary Information

Supplementary Material 1.

Supplementary Material 2.

## Data Availability

The datasets used and/or analyzed during the current study are available from the corresponding author on reasonable request.

## References

[1] Forero M, Adhikary SD, Lopez H, Tsui C, Chin KJ (2016). The Erector Spinae Plane Block: A Novel Analgesic Technique in Thoracic Neuropathic Pain. Reg Anesth Pain Med.

[2] Gao Y, Liu L, Cui Y, Zhang J, Wu X (2022). Postoperative analgesia efficacy of erector spinae plane block in adult abdominal surgery: A systematic review and meta-analysis of randomized trials. Front Med (Lausanne).

[3] Guan HY, Yuan Y, Gao K, Luo HX (2023). Efficacy and safety of erector spinae plane block for postoperative analgesia in breast cancer surgery- A systematic review and meta-analysis. J Surg Oncol.

[4] Nair A, Saxena P, Borkar N, Rangaiah M, Arora N, Mohanty PK (2023). Erector spinae plane block for postoperative analgesia in cardiac surgeries- A systematic review and meta-analysis. Ann Card Anaesth.

[5] Cui Y, Wang Y, Yang J, Ran L, Zhang Q, Huang Q, Gong T, Cao R, Yang X (2022). The Effect of Single-Shot Erector Spinae Plane Block (ESPB) on Opioid Consumption for Various Surgeries: A Meta-Analysis of Randomized Controlled Trials. J Pain Res.

[6] Pawa A, King C, Thang C, White L (2023). Erector spinae plane block: the ultimate 'plan A' block?. Br J Anaesth.

[7] Schwartzmann A, Peng P, Maciel MA, Forero M (2018). Mechanism of the erector spinae plane block: insights from a magnetic resonance imaging study. Can J Anaesth.

[8] Sørenstua M, Zantalis N, Raeder J, Vamnes JS, Leonardsen AL (2023). Spread of local anesthetics after erector spinae plane block: an MRI study in healthy volunteers. Reg Anesth Pain Med.

[9] Shaker EH, Elshal MM, Gamal RM, Zayed NOA, Samy SF, Reyad RM, Shaaban MH, AbdAlrahman AAM, Abdelgalil AS (2023). Ultrasound-guided continuous erector spinae plane block vs continuous thoracic epidural analgesia for the management of acute and chronic postthoracotomy pain: a randomized, controlled, double-blind trial. Pain Rep.

[10] Kurian A, Gallagher S, Cheeyandira A, Josloff R (2010). Predictors of in-hospital length of stay after laparoscopic ventral hernia repair: results of multivariate logistic regression analysis. Surg Endosc.

[11] Chin KJ, Adhikary S, Sarwani N, Forero M (2017). The analgesic efficacy of pre-operative bilateral erector spinae plane (ESP) blocks in patients having ventral hernia repair. Anaesthesia.

[12] Zhang Y, Zhou H, Chai Y, Cao C, Jin K, Hu Z (2014). Laparoscopic versus open incisional and ventral hernia repair: a systematic review and meta-analysis. World J Surg.

[13] The Norwegian medicines manual for health personnel. Ekvipotenstabell for opioider (veilendende ratioer). 2020.[www document] https://www.legemiddelhandboka.no/legacy/chapter/T21.2.1 . Accessed on 10 March 2021.

[14] Wengritzky R, Mettho T, Myles PS, Burke J, Kakos A (2010). Development and validation of a postoperative nausea and vomiting intensity scale. Br J Anaesth.

[15] Pasero C (2009). Assessment of sedation during opioid administration for pain management. J Perianesth Nurs.

[16] Kleif J, Waage J, Christensen KB, Gogenur I (2018). Systematic review of the QoR-15 score, a patient- reported outcome measure measuring quality of recovery after surgery and anaesthesia. Br J Anaesth.

[17] Herdman M, Gudex C, Lloyd A, Janssen M, Kind P, Parkin D, Bonsel H, Badia X (2011). Development and preliminary testing of the new five-level version of EQ-5D (EQ-5D-5L). Qual Life Res.

[18] Stark PA, Myles PS, Burke JA (2013). Development and psychometric evaluation of a postoperative quality of recovery score: the QoR-15. Anesthesiology.

[19] Devlin NJ, Shah KK, Feng Y, Mulhern B, van Hout B (2018). Valuing health-related quality of life: An EQ-5D-5L value set for England. Health Econ.

[20] Efird J (2011). Blocked randomization with randomly selected block sizes. Int J Environ Res Public Health.

[21] StataCorp 2019 (2019). Stata statistical software: Release 16. College Station TSL. Stata Statistical Software.

[22] Bhushan S, Huang X, Su X, Luo L, Xiao Z (2022). Ultrasound-guided erector spinae plane block for postoperative analgesia in patients after liver surgery: A systematic review and meta-analysis on randomized comparative studies. Int J Surg.

[23] Yang X, Zhang Y, Chen Y, Xu M, Lei X, Fu Q (2023). Analgesic effect of erector spinae plane block in adults undergoing laparoscopic cholecystectomy: a systematic review and meta-analysis of randomized controlled trials. BMC Anesthesiol.

[24] Liu J, Fang S, Wang Y, Wang L, Gao L, Xin T, Liu Y (2023). The safety and efficacy of ultrasound-guided erector spinae plane block in postoperative analgesic of PCNL: A systematic review and meta-analysis. PLoS ONE.

[25] Abu Elyazed MM, Mostafa SF, Abdelghany MS, Eid GM (2019). Ultrasound-Guided Erector Spinae Plane Block in Patients Undergoing Open Epigastric Hernia Repair: A Prospective Randomized Controlled Study. Anesth Analg.

[26] Cox B, Durieux ME, Marcus MA (2003). Toxicity of local anaesthetics. Best Pract Res Clin Anaesthesiol.

