# Supplementary material for: Evaluation of the Erector spinae plane block for postoperative analgesia in laparoscopic ventral hernia repair: a randomized placebo controlled trial
Source: BMC Anesthesiol. 2024 May 29;24:192. doi: 10.1186/s12871-024-02566-x (PMC11134963; doi:10.1186/s12871-024-02566-x)
Supplement: Supplementary file 1 — Supplementary Material 1. [file 12871_2024_2566_MOESM1_ESM.pdf]

Table 1: Equipotent table for opioids

Peroral (po) <-> peroral (po)	Ratio
Morphine po : Codeine po	1 : 10
Morphine po : Tramadol po	1 : 10
Morphine po : Oxycodone po	1,5 : 1
Morphine po : Hydromorphone po	5 : 1 til 7,5 : 1
Morphine po : Metadone po	See table 2
**Peroral (po) <-> parenteral (sc/iv)**	**Ratio**
Morphine po : Morphine po sc/iv	3 : 1
Oxycodone po : Oxycodone sc/iv	2 : 1
Hydromorphone po : Hydromorphone sc/iv	2 : 1
**Parenteral (sc/iv) <-> parenteral (sc/iv)**	**Ratio**
Morphine sc/iv : Oxycodone sc/iv	1 : 1
Morphine sc/iv : Hydromorphone sc/iv	5 : 1 til 7,5 : 1
**Peroral (po) <-> transdermal (td)**	**Ratio**
Morphine po : Fentanyl td	100 : 1
Morphine po : Buprenorphine td	75 : 1

Table 2: Equipotent table for metadone

Morphine po mg/day	Ratio Morphine po : Metadone po
30 - 89	5 : 1
90 - 299	6 : 1
300 - 599	8 : 1
600 - 999	10 : 1
