# Supplementary figures and images for: Evaluation of the Erector spinae plane block for postoperative analgesia in laparoscopic ventral hernia repair: a randomized placebo controlled trial
Source: BMC Anesthesiol. 2024 May 29;24:192. doi: 10.1186/s12871-024-02566-x (PMC11134963; doi:10.1186/s12871-024-02566-x)

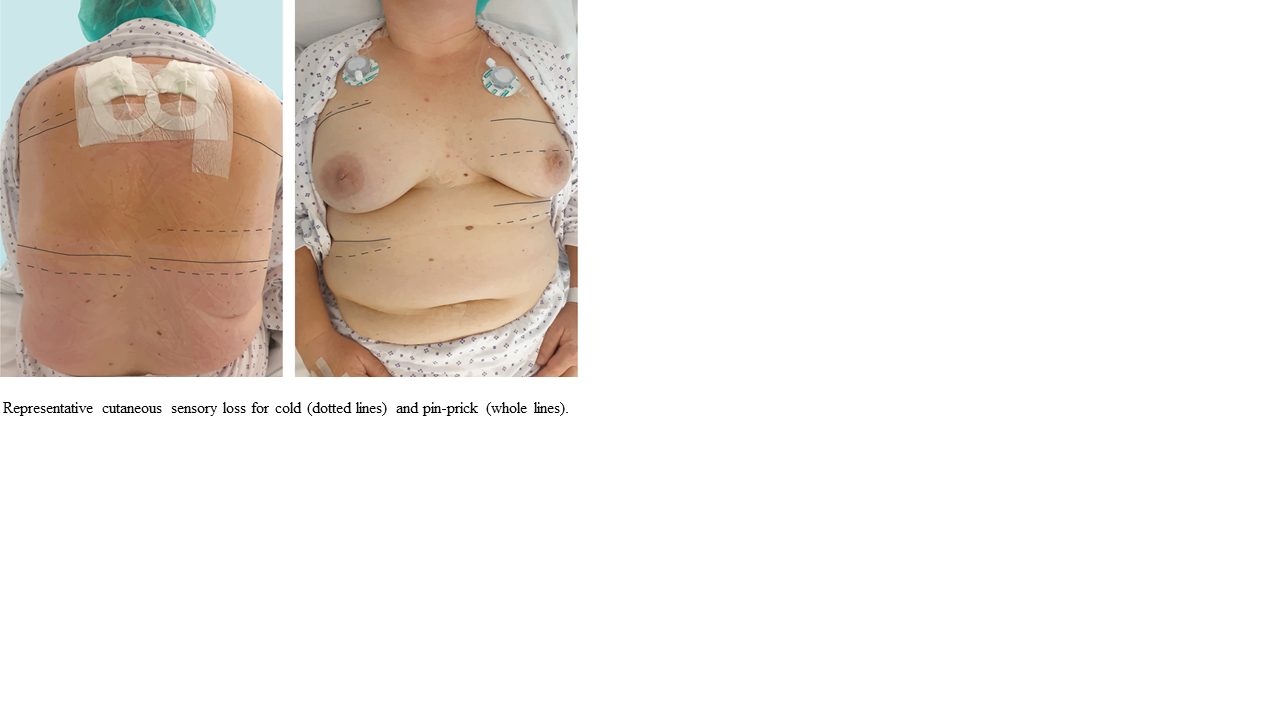

Supplement: Supplementary file 2 — Supplementary Material 2. [file 12871_2024_2566_MOESM2_ESM.tif]
